# Photobiomodulation therapy as an additional method for the treatment of temporomandibular disorder patients– a narrative review

**DOI:** 10.1007/s10103-025-04324-y

**Published:** 2025-01-31

**Authors:** Vesna Karic, Clement Penny

**Affiliations:** 1 Department of Prosthodontic, Laser Therapy in Dentistry Clinic, School of Oral Sciences, Faculty of Health Sciences, WITS University, South Africa; 2 Department of Internal Medicine, School of Clinical Medicine, Faculty of Health Sciences, WITS University, South Africa

**Keywords:** Photobiomodulation, Temporomandibular joint, Temporomandibular disorder, Laser therapy

## Abstract

The photobiomodulation therapy (PBMT) is promising additional therapy in the treatment of temporomandibular disorder (TMD). In this regard, the purpose of this narrative review is to give a wide-ranging, objective, and judicious view of the current knowledge on PBMT as an additional TMD treatment modality, with summarised updated information. Although the results of most research studies report improvement of pain in TMD patients, some state that sustainability of absence of pain after PBMT of TMD is of concern. There has been a recent surge in research around the application of lasers for the management of TMD. Nonetheless, the scarcity of scientific clinical studies with structured laser parameters makes it difficult to draw a more concrete conclusion whether lasers in the treatment of TMD are more effective than traditional TMD treatments. In conclusion, since PBMT is becoming an additional treatment of choice for the management of TMD there is a need for more research especially involving clinical studies with better structured laser parameters.

## Introduction

**Fig. 1:**
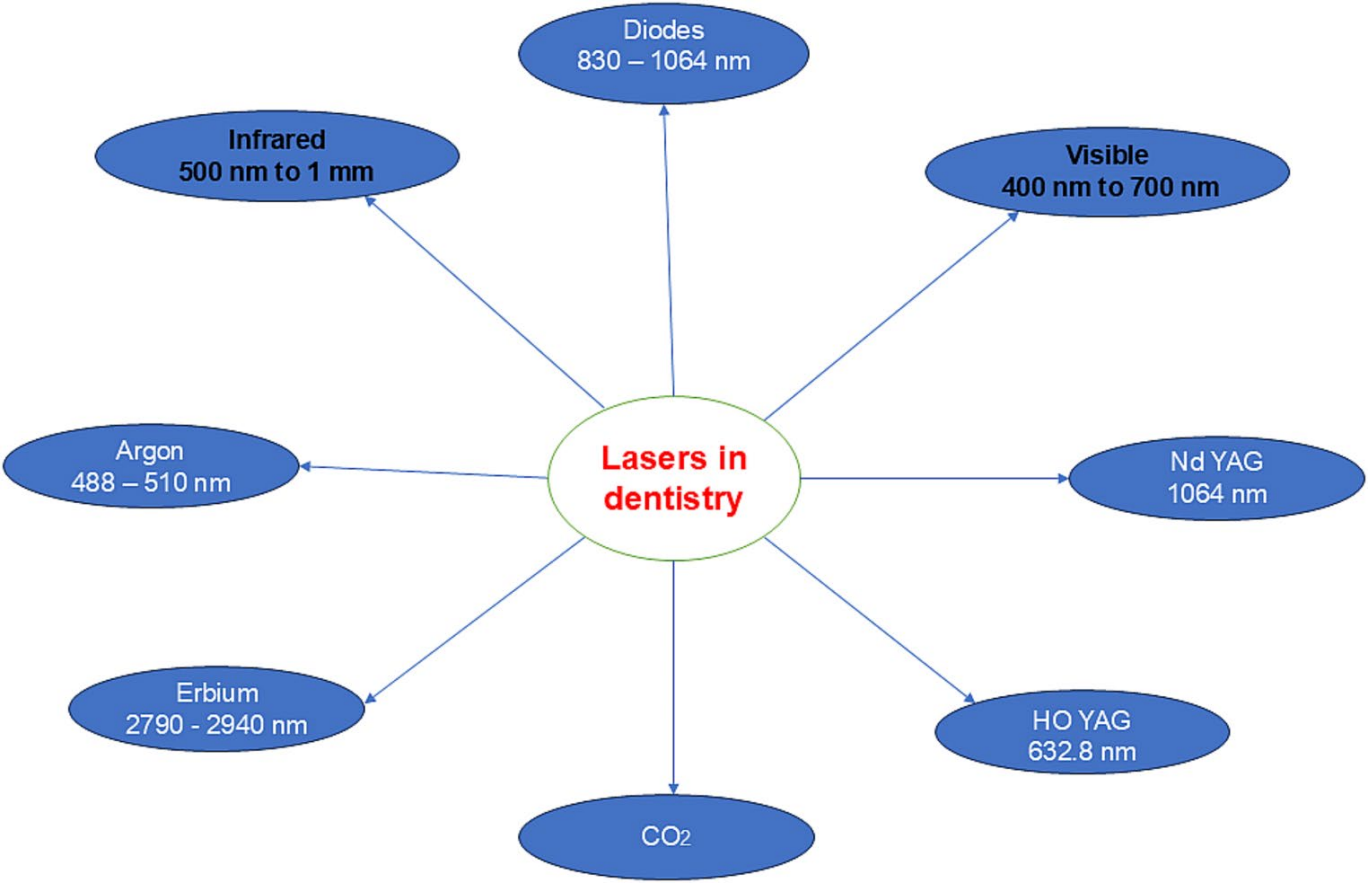
The classification of lasers in dentistry

Laser therapy has evolved as an additional therapy in dentistry in recent years, stimulating/triggering the interest of many researchers and clinicians. Today dental lasers are classified according to laser affinity to tissues and wavelength, and into low and high output lasers and according to the lasing medium [1], (see Fig. 1 as a summary).

In general dentistry lasers have been used as an additional treatment in the field of orthodontics, implants, prosthodontics, periodontics, and other dental fields [2]. On the other hand, laser therapy is only starting to become known as an additional treatment for temporomandibular disorder (TMD) (disorder of TMJ and its muscles). Lasers are gaining momentum in the treatment of TMD due to its non-invasive treatment attributes, giving patients a promising future therapeutic course of therapy for TMD. In addition, the non-invasive aspect of TMD laser treatment provides advantages as the treatment is reversible and even offers possible improvement in psychological and emotional symptoms connected to TMD [3].

Likewise, laser treatment is effective in relief of pain in myogenic and arthrogenous TMD patients and to date has not resulted in adverse reactions declaring laser treatment of TMD commonly clinically safe [4]. The treatment of TMD has a purpose of removing pain and reinstating temporomandibular joint (TMJ) function [3, 5].

Temporomandibular disorder is the furthermost present long-lasting, non-dental related, discomfort condition in the dental populace.

### Signs/symptoms of temporomandibular disorder

It has been reported that approximately 33% of the overall populace displays more than one sign or symptom related to TMJ, of which approximately 5% will actively seek treatment, with female patients predominating in most patient groups [6, 7]. In addition, delayed reporting of the TMD patients for treatment presents a significant challenge as relaxation techniques and stabilization splints are mostly dependent on patient compliance and do not offer instant pain relief [8]. Conversely, a low-level laser therapy (LLLT) has been applied as a treatment of the TMD patients to lower inflammation and for pain treatment [9–11]. Here, photobiomodulation therapy (PBMT) is the preferred and most current term used [12, 13]. Hence, the PBMT of TMD gives new hope and opens a new window of opportunity enhancing TMD treatment in the future.

The TMD patients are categorized into two broad diagnostic groups, the myogenous and arthrogenous TMD groups [14, 15]. The complex list of symptoms reported by the TMD patients includes headache, ear pain, tinnitus, dizziness, lack of sensation in different parts, allodynia, chronic tiredness; and sleep disorders [16, 17]. Additionally, dysmenorrhea and irritable bowel syndrome (IBS) have also been presented as a part of the TMD patient’s clinical signs [18]. The studies have confirmed the association between headache and TMD and that a major proportion of these patients suffer from headaches as related to the overall populace [19–22]. Though, the studies have confirmed the presence of the headaches and TMD without any connection as a separate health issue [23, 24]. In addition, it has been reported that motor vehicle accident victims report TMD symptoms inclusive of headaches [25]. As well, some studies reported that association of headaches with TMD was dependent upon the nature of headache. Furthermore, migraines have been linked at 7% with TMD, and 46% are tension headaches connected to TMD patients’ clinical picture according to research studies [26].

However, it has been reported that TMD patients with concomitant headaches and pain, had recovery in both signs and symptoms after TMD treatment [27]. Also, the medical conditions, headaches, earaches, neck pain and other head and neck symptoms could be interlinked with all TMD the cardinal signs and symptoms (pain in joint and/or muscles, joint sounds, and limitation of movement) [28]. Hence, this multifactorial etiology of the TMD creates the management of TMD difficult. Therefore, the Research Diagnosis Criteria for Temporomandibular Disorders (RDC/TMD) were corroborated to show the universal standards for evaluation of TMD with *the dual axis system (assessment of both clinical signs and symptoms (Axis I) and the biobehavioural domain (Axis II)) application* [29, 30].

### Temporomandibular disorder etiology

**Fig. 2:**
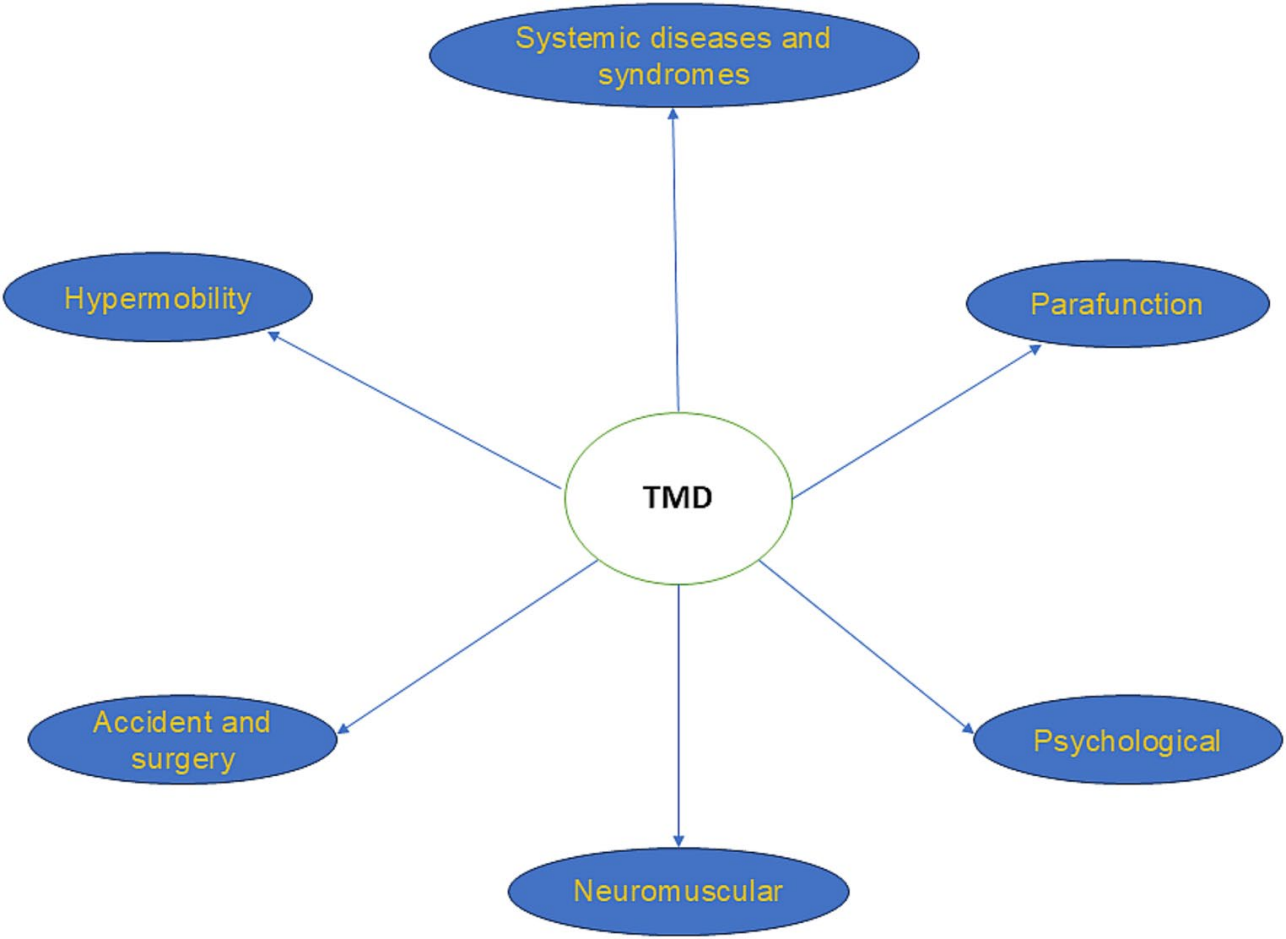
Temporomandibular disorder complex - multifactorial etiology

The multifactorial etiology of TMD patients makes management of TMD difficult, partly because of the different origins of etiology. The etiological factors origin is very often a confusing clinical picture of TMD, because the origin could be either in TMD disorder or systemic diseases (arthritic, increased oestrogen, stress, anxiety (acute TMD), depression (chronic TMD)), or both. In addition, the etiology of TMD patients could be interlinked with syndromes, and the etiology of TMD patients’ health issues could be of genetic origin. For example, the hereditary disorder Marfan and Ehler–Danlos syndromes are associated with TMJ and related to the biochemical irregularity concerning fibrous matrix proteins. In addition, the biochemical incidents (inflammatory contributors) related to TMD patients’ etiology have been researched by only a few research studies, Fig. 2 [31].

The biochemical profiles of inflammatory contributors to TMD are of extreme importance and might be underlining factors that the treatment of TMD is overseeing. The hidden biochemical profiles could be the reason for failure of TMD treatment. For example, the aquaporins (AQPs) with specificity of the AQP1 are part of normal TMJ structures (TMJ disc - fibrocartilage - membrane channel proteins) and they are part of the normal TMJ disc physiological mechanisms involved with tissue homeostasis. The suggestion of this research outcome was that future research studies should investigate the role of AQP1 in unhealthy TMJ discs as part of the treatment of the TMD patients [31].

Therefore, the treatment of TMD becomes complex and difficult, and it involves many disciplines.

### Treatment of temporomandibular disorder

The two universal methods of TMD treatment for patients are stabilization splint otherwise called bite plate or occlusion (occlusal splint) and relaxation therapy. The research that analyzed treatment of TMD patients with occlusion splints in thirty-three TMD patients during the four weeks of treatment with splint had twenty-eight patients returning with improved pain scores and twenty-seven with increase in maximal interincisal distance. However, the upturn in maximum mandibular opening was not statistically significant [32]. A variation of the Jacobson’s relaxation technique was developed for the management of TMD patients in the year 1968 [33]. Furthermore, the study reported a substantial decline in pain and recovery with jaw function in patients with myofascial pain using counseling and physical treatment [34]. In addition, the research results report that occlusal splint and laser auriculotherapy generated related outcomes for the TMD patients’ treatment with bodily and mental aspects of the TMD patients. For example, the improvements were recorded when occlusal splint was used in muscle pain (left temporal), masseter muscle pain (left and right), joint pain(left), and lateral pterygoid muscle. The laser auriculotherapy group gave similar results but with slight differences (improvements in the function of the jaw, pain in left masseter, TMJ pain (left and right), and lateral pterygoid muscle pain (left and right). Possible due to the application of laser to the auricular acupoints that upholds analgesic blockade [35].

Hence, the emergence of PBMT as an additional treatment option has given more hope for better and more definite treatment of TMD.

## Discussion

This narrative review is projecting a wide-ranging, objective, and judicious view of the current knowledge on PBMT as an additional method for the treatment of TMD.

The possibility of having an additional PBMT method as a cost-effective treatment is very important to the patients who suffer from TMD. The PBMT as non-invasive treatment offers advantages to the TMD patients as the treatment diminishes even the psychological aspects related to the TMD, and as reported by the systematic review study [3]. Similarly, the PBMT has been declared by studies as an additional and safe non-invasive method of treatment for TMD patients.

Nevertheless, the systematic review study reports discrepancies with the laser delivery parameters, frequency of sessions, and laser intervention sites of PBMT in TMD patients and gives indication of a need for more studies with randomized clinical trials, and with set parameters and dosages to establish itself as a confirmed additional TMD treatment. Hence, the absence of methodological appropriate processes in the clinical studies confines the indication of the success of PBMT as a TMD treatment and inhibits the possibility of creating a foundation for the precise dose and favourable laser parameters for PBMT for the treatment of the TMD patients [36].

Furthermore, the World Association of Laser Therapy consensus (2004) has established a rule for the plan of clinical studies with application of PBMT in TMD patients where the study design requires a placebo group. The systematic review study examined studies in application of this rule and established that most PBMT treated TMD groups were compared to the placebo groups. However, seven studies did not include placebo groups which is concerning. The results of the seven studies could be declared Nula void compared to appropriate methods from the studies that applied placebo groups in their studies. As well, this systematic review established that laser wavelengths varied between 632.8 nm and 1064 nm and that wavelengths less than 780 nm were only applied in five studies. In addition, the results did confirm variety in the laser parameters, including different dosage, variety of the power outputs (1 mW up to 10 W), and different range of the time of irradiation with pulsed or continuous beams and using a range of 30–60 s per point of application. In conclusion it was confirmed that the deliberation on the usefulness of PBMT in the treatment of TMD patients was caused by the wide variety of the laser dosage used for treatment of TMD resulting in unsustainable inconsistency [37]. Thus, the differences in methods in the various studies brings all PBMT results in the treatment of TMD in question and opens further debate on what is appropriate as the PBMT treatment of the TMD patients.

Likewise, another study that treated myogenic TMD in the group of twenty female patients applying randomized, double-blind clinical trial rules applied a pulsed 810-nm laser, whereby they randomly divided patients into laser and placebo groups. This study used a pulsed 810-nm laser at 50 mW (standard power) and applied a peak power of 80 W at 1,500 Hz for duration of 120 s and at 6 J, and per 3.4 J/cm^2^ for each painful point, for the management of TMJ muscular pain. The treatment frequency was three times per week for a duration of four weeks and similarly with the placebo group, but without laser output application. In this study the management of myogenic TMD applying a pulsed 810-nm laser produced a notable progress in mouth opening, with reduced pain levels in myogenic TMD patients. Then again, no statistical analysis was done to compare the laser treated groups with placebo groups, regarding pain intensity and actions of the mandible. Moreover, all the improvements in the symptoms of the myogenic TMD patients described in this study (pain, mouth opening) in the laser group had a limited duration of only 1 month after the last treatment application [38]. The short-lasting relief of symptoms in TMD patients post treatment with PBMT is of great importance and even greater concern. As a previous study example this study method as well opens debate on how further studies should be formatted to ensure accurate results for PBMT in the treatment of the TMD.

Conversely, it is the general conclusion that PBMT successfully relieves pain for the TMD patients as an immediate effect compared to transcutaneous electric nerve stimulation (TENS) [39]. In addition, the laser’s ability to decrease and prevent bone tissue regeneration after ablation have been explored with surgical procedures [40]. The PBMT in the study that used 910 nm to 1100 nm wavelength reported superior TMD related pain-relieving effects for the short term [41]. As well, it has been recommended that PBMT could be used as laser acupuncture therapy (LAT) with laser given better results compared to acupuncture treatment [42]. Another study using a 780–980 nm wavelength infrared diode laser, for approximately six sessions of treatment, has given hopeful choices for the TMD treatment. The numerous parameters have a crucial role in the importance of specific PBMT protocols for treating TMD, the PBMT could give a hope for better future designs and standardization of the protocols [43]. In another study it was concluded that both treatment with occlusal splint and PBMT applied in the management of myofascial pain syndrome are successful, and with thermal images data, PBMT gave promising offers for treatment of TMD patients [44, 45]. The importance of new additional treatment for TMD becomes imperative when the TMD influence on patient quality of life is observed and severity of that influence [46].

The present endorsements of laser therapy as an additional treatment for TMD patients could be summarized to recommend diode laser type for various TMD patients and arthrogenous TMD patients’ treatment may be done with 400 and 800 nm range of wavelength. A wavelength between 800 and 1500 nm for patients with myogenous TMD, looks promising and with dosage of < 25 J/cm^2^. While in the TMD patients with mixed symptoms, the dosage indicated is > 100 J/cm^2^, at 15–30 s or > 60 s application. Therefore, the conclusion regarding which PBMT management modality must be engaged can be based on the TMD patient’s symptoms [47]. On the contrary, the study that evaluated the effect of PBMT on TMD patients in pain using placebo-controlled manners applied a randomized, double-blinded method indicating a substantial non-specific effect of PBMT [48]. Compared to previous studies methods reported in this section, this study followed all appropriate research methods and resulted in a non-specific effect of PBMT on TMD patients. For this reason, the future research studies should examine more variety of laser parameters, treatment routines, different valuation time frames, and different consequence measures [49].

However, most studies have explored lasers in different laser parameters and in remarkably diverse settings, bringing confusion to selection of lasers for the treatment of the TMD patients. In addition, it is concerning that most studies give short term results and no follow-up results which then weaken the impact of PBMT on TMD treatment. The most favorable output in the management of TMD patients with PBMT would be a permanent solution and no TMD.

### Concluding remarks and recommendations

Photobiomodulation therapy as an alternative in the management of TMD has arrived as a novel additional treatment. The mostly used lasers for the treatment of the TMD patients are between 632.8 nm and 1064 nm wavelengths with 810 nm and 910 nm lasers in most frequent application. There has been a recent surge in research around the practice of application of PBMT in the treatment of TMD with very little exploration in the influence of PBMT. However, the need for standardization of future research studies with widening of the laser spectrums and better standard of parameters for the purpose of providing a better additional treatment option to TMD patients is evident. Additionally, there is demand for more longitudinal research studies with extensive follow up time frames.
